# Transcriptomics- and Genomics-Guided Drug Repurposing for the Treatment of Vesicular Hand Eczema

**DOI:** 10.3390/pharmaceutics16040476

**Published:** 2024-03-30

**Authors:** Fieke M. Rosenberg, Zoha Kamali, Angelique N. Voorberg, Thijs H. Oude Munnink, Peter J. van der Most, Harold Snieder, Ahmad Vaez, Marie L. A. Schuttelaar

**Affiliations:** 1 Department of Dermatology, University Medical Center Groningen, University of Groningen, 9713 GZ Groningen, The Netherlands; f.m.rosenberg@umcg.nl (F.M.R.); a.n.voorberg@umcg.nl (A.N.V.); 2 Department of Epidemiology, University Medical Centre Groningen, University of Groningen, 9713 GZ Groningen, The Netherlandsh.snieder@umcg.nl (H.S.); 3 Department of Bioinformatics, School of Advanced Medical Technologies, Isfahan University of Medical Sciences, Isfahan P.O. Box 81746-7346, Iran; 4 Department of Clinical Pharmacy and Pharmacology, University Medical Center Groningen, University of Groningen, 9713 GZ Groningen, The Netherlands; t.h.oude.munnink@umcg.nl

**Keywords:** vesicular hand eczema, hand eczema, transcriptomics, genomics, drug repurposing, treatment

## Abstract

Vesicular hand eczema (VHE), a clinical subtype of hand eczema (HE), showed limited responsiveness to alitretinoin, the only approved systemic treatment for severe chronic HE. This emphasizes the need for alternative treatment approaches. Therefore, our study aimed to identify drug repurposing opportunities for VHE using transcriptomics and genomics data. We constructed a gene network by combining 52 differentially expressed genes (DEGs) from a VHE transcriptomics study with 3 quantitative trait locus (QTL) genes associated with HE. Through network analysis, clustering, and functional enrichment analyses, we investigated the underlying biological mechanisms of this network. Next, we leveraged drug–gene interactions and retrieved pharmaco-transcriptomics data from the DrugBank database to identify drug repurposing opportunities for (V)HE. We developed a drug ranking system, primarily based on efficacy, safety, and practical and pricing factors, to select the most promising drug repurposing candidates. Our results revealed that the (V)HE network comprised 78 genes that yielded several biological pathways underlying the disease. The drug–gene interaction search together with pharmaco-transcriptomics lookups revealed 123 unique drug repurposing opportunities. Based on our drug ranking system, our study identified the most promising drug repurposing opportunities (e.g., vitamin D analogues, retinoids, and immunomodulating drugs) that might be effective in treating (V)HE.

## 1. Introduction

Hand eczema (HE) is a multifactorial inflammatory skin disease that can be classified into the etiological subtypes allergic contact dermatitis, irritant contact dermatitis, atopic HE, or protein contact dermatitis. Another classification approach is based on clinical subtypes: hyperkeratotic HE, acute recurrent vesicular HE (VHE), nummular HE, and pulpitis [1]. HE is a common skin disease with a lifetime prevalence of 15% [2,3]. In a cross-sectional questionnaire-based study, 2% of the Dutch general population reported ever having experienced severe HE [2]. Chronic HE, especially with increased severity, can cause a significant burden in patients as a result of functional impairment, a decline in quality of life, and reduced work ability. Additionally, chronic HE places a substantial economic burden on society due to frequent health care utilization, high rates of sick leave, and job loss [4,5,6,7,8]. Despite its high prevalence and burden, effective treatment options for long-term control of chronic HE remain limited [1,9].

Currently, the only approved systemic treatment option in the European Union for severe chronic HE refractory to (very) potent topical corticosteroids is the retinoid alitretinoin, an oral vitamin-A derivative [1,9,10]. According to a Cochrane review, 44% of severe HE patients treated with alitretinoin achieved almost clear to clear hands compared to 16% in the placebo group [10,11,12]. Moreover, the effectiveness of alitretinoin depends on the subtype of HE, as patients were more likely to be responsive to alitretinoin in hyperkeratotic HE (49% achieved clear to almost clear) compared to VHE (33%) [11]. Common adverse events are headaches, increased cholesterol and triglyceride levels, and a decrease in thyroid function [1]. Consequently, there is a large unmet need for HE patients who are unresponsive or intolerant or when alitretinoin is medically inadvisable, [1,9,10]. The high prevalence of HE, the substantial number of severe HE cases, the significant burden, and lack of effective systemic treatment options, emphasize the need for new or alternative treatment approaches.

A recently published transcriptome and proteome analysis of VHE has highlighted similarities with the transcriptome and proteome of atopic dermatitis (AD), suggesting that treatments for AD may also be effective for VHE [13,14]. Drug repurposing could be a useful tool for identifying existing alternative treatment options for HE, such as approved treatment options for AD [15].

The aim of our study is to identify drug repurposing opportunities for VHE using transcriptomics and genomics data. First, a network was constructed by combining 52 differentially expressed genes (DEGs) from the VHE transcriptome [13] and 3 quantitative trait locus (QTL) genes associated with HE [16]. Next, through network analysis, clustering, and functional enrichment analyses, the underlying biological mechanisms of our network were attempted to be uncovered. Drug–gene interactions were leveraged in the DrugBank database [17] to identify drug repurposing opportunities for (V)HE. Finally, a drug ranking system was developed to select the most promising drug repurposing candidates for treatment of (V)HE.

## 2. Materials and Methods

### 2.1. Network Construction

A network was constructed from three sources. First, a list of 52 DEGs was derived from our previously published paper, which compared transcriptomic signatures of ten lesional palmar VHE skin samples to ten healthy control palmar skin samples [13]. Second, the results of our GWAS of self-reported HE in lifetime (unpublished data) [16], encompassing all clinical HE severities, were obtained from the Dutch general population within the Lifelines Cohort Study [18]. Subsequently, the genotype-tissue expression (GTEx) database [19] was queried with the most significant variant from the GWAS (20q13.33, rs6011058) [16], seeking effects on splicing or expression level of genes (namely, splicing/expression QTL; s/eQTL) in sun-exposed and not-sun-exposed skin tissue. Third, the Reactome functional interaction plugin version 8.0.5 [20] for Cytoscape [21] was used to add the minimal required additional genes as linkers and create a single connected network.

### 2.2. Network Analysis and Clustering

The constructed combined network was analyzed with Cytoscape version 3.9.1 [21] joined with NetworkAnalyzer version 4.4.6. Centrality parameters, including degree for nodes and betweenness for connections, were investigated to flag hub genes and connections that were crucial for the disease network survival. Spectral partition based network clustering [22], provided by the ReactomeFI Cytoscape plugin, was applied to find specific functional modules throughout our constructed combined network.

### 2.3. Functional Enrichment Analyses

Reactome pathway enrichment analysis was applied separately on (1) the 52 DEGs of VHE, (2) 3 QTL genes of the HE GWAS locus, (3) the whole constructed combined network, and (4) the individual identified modules. Additionally, the Cytoscape STRING plugin version 2.0.1 [23] was used to perform extensive functional enrichment analyses for our combined network across different functional categories, including protein domains [24,25], protein families [26], protein clusters [27], cell compartments [28], tissues [29], gene ontologies [30], biological pathways (Reactome [31] and KEGG [32]), diseases [33], Monarch phenotypes [34], and UniProt keywords [35]. As a sensitivity analysis, the enrichment analysis was repeated by filtering the network to nodes with a higher-than-average skin tissue expression (STRING score > 2.5 in skin).

### 2.4. Drug–Gene Interactions

First, all genes in our network, including the 52 DEGs from the previous VHE paper [13], 3 QTL genes of the HE GWAS locus, and the 23 added linker genes, were used as query in the DrugBank database (version 5.1.10, 4 January 2023) [17] to find possible drugs that target genes within our (V)HE network. Second, DrugBank pharmaco-transcriptomics data of the 52 DEGs were retrieved in order to find an opportunity for reversing the VHE transcriptomic profile to a healthy status using currently available approved drugs. The last step was to check pharmaco-genomics of the loci identified in our HE GWAS analysis through the same database. Not only was the index single-nucleotide polymorphism (SNP) of the GWAS loci used but also all genome-wide significant (*p*-value < 5 × 10^−8^) SNPs in order not to lose SNP–drug interactions that were reported for a relevant but nearby SNP in the region.

### 2.5. Drug Ranking System

To identify the most promising drug repurposing candidates, a drug ranking system was developed based on expert opinions of a clinical pharmacist and dermatologist. Our drug ranking system included several bonus and penalty criteria, which were primarily based on efficacy, safety, pricing, and practical factors (Table 1). A manual literature search for every drug repurposing candidate was performed using the websites ClinicalTrials.gov [36], PubMed [37], the World Health Organization (WHO) list of essential medicines in 2023 [38], ‘Royal Dutch Pharmacists Association Kennisbank’ [39], the WHO ATC index [40], and ‘Farmacotherapeutisch Kompas’ [41] to assign the bonus and penalty points, resulting in a final score for each drug candidate (Table 1).

Bonus points were given to the drugs that showed immunomodulating efficacy in auto-inflammatory and auto-immune (skin) diseases in a completed phase 2 interventional trial and if these drugs were at least investigated in an ongoing phase 3 trial according to ClinicalTrials.gov [36] or PubMed [37]. Bonus points were also assigned to drugs that target more than three genes of our (V)HE network as we thought that drugs that target more genes may be more effective compared to drugs that target fewer genes. Drugs that were included in the WHO list of essential medicines in 2023 [38] received a bonus point as this indicates global drug accessibility. Drugs that could be applied topically or could be compounded to a topical application (availability of a raw resource, suspension, injection powder, excluding biologicals) received bonus points according to the ‘Royal Dutch Pharmacists Association Kennisbank’ [39]. The local concentration of topical application could minimize systemic side effects.

Penalty points were given to the drugs with no Anatomical Therapeutic Chemical (ATC)-code according to the WHO ATC index [40] and if a drug was not available to order in the Netherlands according to the ‘Royal Dutch Pharmacists Association Kennisbank’ [39]. Drugs with an unclear direction of effect on genes were given a penalty point, e.g., when the direction of effect was not available. Moreover, if a drug had a 0.1–1% chance on a grade 3–5 adverse event (severe including hospitalization or disabling, life-threatening, or death [42]) according to ‘Farmacotherapeutisch Kompas’ [41], the drug received a penalty point for safety reasons. Furthermore, a drug was given penalty points when it contained practical issues (e.g., when the route of administration was only parenteral intravenous) and severe practical issues (e.g., when the drug formulation was gas or skin substitute).

The price of the drug was retrieved from ‘Farmacotherapeutisch Kompas’ [41], a Dutch website from the government offering independent drug information for healthcare professionals. The price was converted to the annual costs in euros. Bonus points were awarded for cheap (50–500 EUR/year) and very cheap (<50 EUR/year) drugs, while penalty points were assigned when the price was unavailable or very expensive (>20,000 EUR/year, i.e., above the current pricing range for biologicals in the treatment of atopic dermatitis). Finally, as secondary analyses, we repeated the drug ranking without pricing criteria to select the most promising candidates without the influence of cost considerations.

## 3. Results

### 3.1. Network Construction

**Figure 1:**
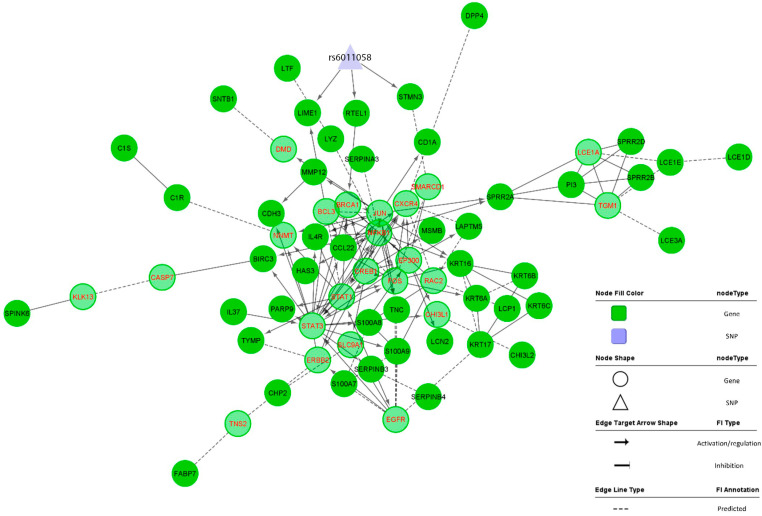
Combined network of the 52 vesicular hand eczema differentially expressed genes, 3 quantitative trait locus genes from the hand eczema locus (presented with black labels), and 23 linker genes (presented with partly transparent fill color and red labels). Interactions with no specified direction e.g., complex formation, are shown as simple lines without an arrow at the edge.

The (V)HE combined network, depicted in Figure 1, was constructed using the 52 DEGs from the transcriptome analysis of VHE [13], 3 genes from QTL lookups of the HE GWAS locus [16] (Table S1 and Figure S1), and a total of 23 genes added by the ReactomeFI Cytoscape plugin as linkers. No functional interaction data could be found for nine genes from our input list, i.e., *CD207*, *HEPHL1*, *PRSS53*, *S100A7A*, *TMEM173*, *AADAC*, *C5orf46*, *LOR*, and *MT4*.

### 3.2. Network Analysis and Clustering

**Figure 2:**
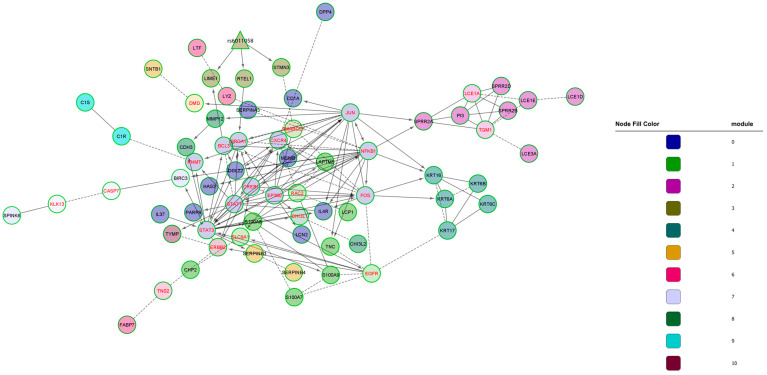
Clustered network of the 78 (vesicular) hand eczema genes with 11 identified modules shown in different colors.

Various centrality parameters were calculated for genes and interactions, and the full results are presented in Tables S2 and S3. The degree of centrality for the network nodes followed the power-law distribution, suggesting that the constructed network is a true biological network, not a random one [43] (Figure S2). *STAT3*, *NFKB1*, *JUN*, *STAT1*, *FOS*, *EP300*, and *CREB1* were the top 10% most central genes in the (V)HE combined network, identified by the degree parameter (Table S2). Interactions of *NFKB1* with *SPRR2A*, *LYZ*, *RAC2*, *BIRC3*, and *STAT3*, as well as interactions of *STAT3* with *BIRC3* and *SERPINB3*, were the most crucial for the disease network as identified by betweenness centrality parameter (top 10%), likewise were the interactions between *JUN* and *DMD* or *SPRR2A* and between *SPRR2A* and *TGM1* or *LCE1A*. Other highly connective interactions included *BIRC3-CASP7*, *CASP7-KLK13*, *C1R-NNMT*, and *ERBB2-TNS2* (Table S3). Clustering of the combined network by CytoScape/NetworkAnalyzer resulted in 11 modules, depicted in Figure 2.

### 3.3. Functional Enrichment Analyses

Functional enrichment results of separate analyses of (1) the 52 DEGs of VHE, (2) 3 QTL genes of the HE GWAS locus, (3) the combined (V)HE network, and (4) the 11 modules are presented in Tables S4–S6. In summary, keratinization, cornified envelope formation, antimicrobial peptides, and immune response were among the most significant findings. Keratinization and innate immune system involved the highest numbers of genes from our 52 VHE DEGs (i.e., 13 and 14 genes, respectively, in Table S4A), and from our combined (V)HE network (i.e., 16 and 21 genes, respectively, in Table S5). Telomere maintenance was the most frequent term amongst functions enriched for HE QTL genes (Table S4B).

Four out of the eleven identified modules were (partly) involved in keratinization or keratinocyte differentiation (Table S6). Separate diagrams of some of the important pathways, including keratinization, immune responses, and antimicrobial peptides with nodes involving our 55 (V)HE genes highlighted in a different color, are presented as Figures S3–S5.

Enrichment analyses across 12 different databases through STRING returned 177 significant terms at false discovery rate < 0.05 (Table S7). Among these, keratinization in UniProt keywords and STRING clusters, as well as keratinocyte differentiation in the GO biological process, were the most striking and expected findings. Furthermore, the intermediate filament protein (e.g., keratin) was the most significant term in protein families and SMART domains. In line with these and as part of the skin barrier, the cornified envelope was the most significant cellular compartment. Similarly, the most significant term in InterPro domains was the small proline-rich protein/late cornified envelope protein. The stratum spinosum was the most significant term in functional enrichment of the category ‘tissues’, which is the second skin layer of the epidermis above the stratum basale, which mainly consists of keratinocytes held together by desmosomes with a crucial role in keratinization. [44]. Additionally, skin-related disease and phenotypes involved in keratinization were the most significant terms across the categories ‘diseases’ and ‘Monarch phenotype’, such as pachyonychia congenita in the category ‘diseases’ and linear arrays of macular hyperkeratoses in flexural areas in the category ‘Monarch phenotype’. Finally, the most significant term in GO molecular function was endopeptidase inhibitor activity, and the most significant term in KEGG was the IL-17 signaling pathway.

A sensitivity analysis using only the 47 genes with above-average STRING scores of skin expression (>2.5) returned highly consistent results, with 84% of the significant terms repeated, including the most significant terms of each category (Table S8).

### 3.4. Drug–Gene Interactions

**Figure 3:**
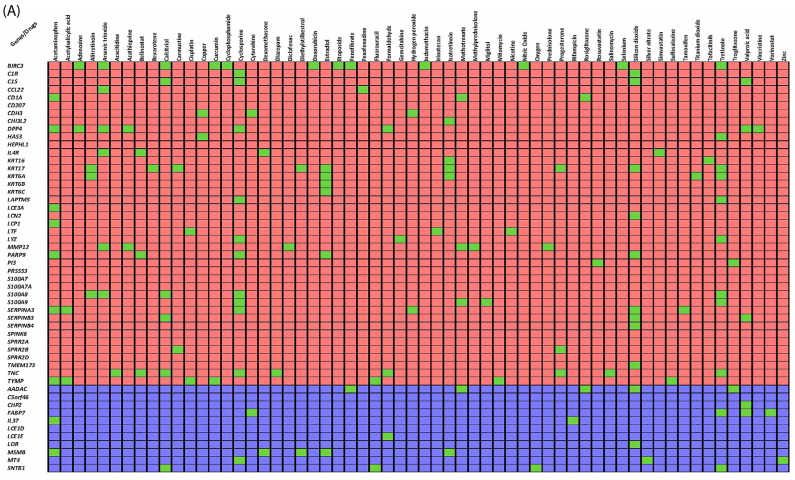

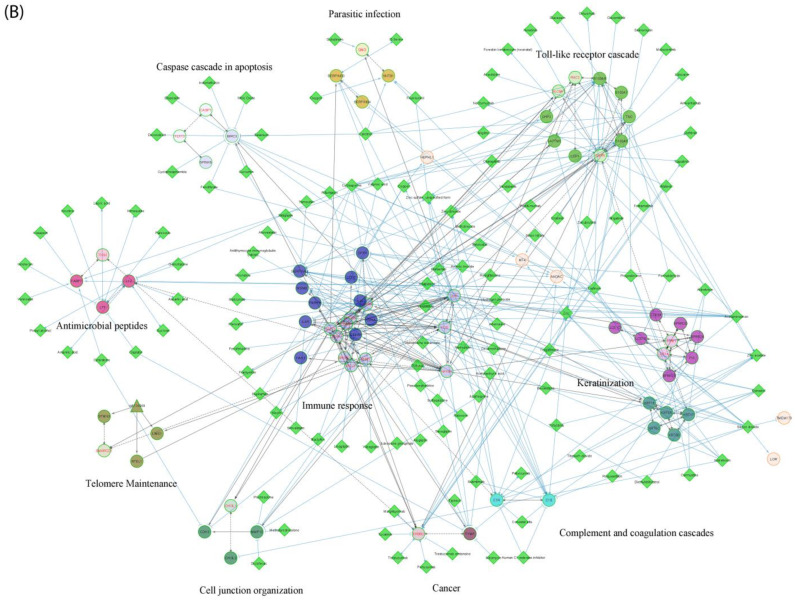
Drug–gene interactions of (vesicular) hand eczema ((V)HE) genes based on the DrugBank database. (**A**) Pharmaco-transcriptomic lookups of 52 VHE DEGs (red and blue cells indicate up/downregulated genes; green cells indicate gene expression changes of the drugs opposing those of VHE. (**B**) The combined (V)HE gene network classified in the different functional modules with general functions annotated. The interacting drugs (physical interaction or gene expression regulations) targeting these functional modules with a general function are shown as green diamonds.

A drug–gene interaction search of our 78 genes in the (V)HE network (i.e., 52 VHE DEGs + 3 HE QTLs + 23 linkers) in DrugBank returned 67 unique drugs targeting 16 main genes and 9 linkers (Table S9). Pharmaco-transcriptomic lookups revealed 60 drugs which can partly reverse the transcriptomic changes that occur in VHE disease (Table S10, Figure 3A). The results showed that the drugs tretinoin, cyclosporine, and silicon dioxide affected the most VHE DEGs, reversing the gene expression of 11 VHE DEGs to a normal level. In combination with each other, they can potentially reverse the gene expression of a total of 23 VHE DEGs to the normal level (Table S10). Pharmaco-genomics investigations of the genome-wide significant SNPs from our HE GWAS did not highlight any drugs. In total, we identified 123 unique drug repurposing candidates for the treatment of (V)HE, all visualized in Figure 3B.

### 3.5. Drug Ranking System

Our drug ranking system revealed a list of the most promising drug repurposing candidates for the treatment of (V)HE with a score > 0 (Table 2), including vitamin D analogues, retinoids, immunomodulators, hormones, antihistamines, antibiotics and antibacterials, vitamins and minerals, and cardiovascular, blood glucose-lowering, antineoplastic, non-steroidal anti-inflammatory, and nervous system-modulating drugs. The full list of all drug repurposing candidates with assigned points according to the drug ranking system is presented in Tables S11–S13. In the secondary analysis, the most promising drug repurposing candidates were selected without considering the pricing criteria. The drugs irbesartan, rosuvastatin, saxagliptin, and silicon dioxide had their scores changed from 1 or 2 to 0 when pricing criteria were excluded. As a result, these drugs were no longer considered as promising drug repurposing opportunities (Table S13). These drugs had been initially included mainly based on their low to very low pricing, rather than other criteria.

## 4. Discussion

In this study, a (V)HE network was constructed of 78 genes, including 52 VHE DEGs, 3 HE QTL genes, and 23 linker genes. The pathways keratinization, cornified envelope formation, antimicrobial peptides, and immune response were the most significant findings in our gene network. The drug–target interaction search in DrugBank [17] together with pharmaco-transcriptomic lookups revealed 123 unique drugs as potential drug repurposing opportunities for (V)HE. Based on our drug ranking criteria, we identified the most promising drugs as repurposing opportunities for the treatment of (V)HE. The potential of several of these most promising drugs, classified in medicine groups, is explained below.

### 4.1. Vitamin D Analogues

One of the most promising drug repurposing opportunities for (V)HE is calcitriol, an active oral and topical vitamin D3 analogue. Topical vitamin D3 analogues are widely used to treat psoriasis as they can reduce keratinocyte proliferation, induce keratinocyte differentiation, and have immunomodulating effects [45,46,47]. A more potent synthetic vitamin D3 derivative is topical calcipotriol [45,46,48]. Calcipotriol also showed efficacy in various dermatological diseases, like actinic keratosis, alopecia areata, lichen sclerosus, vitiligo, lichen planus, and palmoplantar hyperkeratosis [46,49,50]. Furthermore, calcipotriol ointment compared to a potent topical corticosteroid in an intraindividual right–left randomized controlled trial (RCT) demonstrated comparable clinical improvement (up to 76%) in chronic mild-to-moderate HE patients (10 hyperkeratotic HE, 2 pulpitis, and 1 VHE) after 8 weeks [51].

### 4.2. Retinoids

Several retinoids (alitretinoin, isotretinoin, adapalene, tretinoin, and bexarotene) were selected as promising drug repurposing opportunities for (V)HE. Retinoids influence the biological processes of keratinization, cornified envelope, and immune response, which were also the most significantly enriched processes among the genes in our whole (V)HE network [52,53]. Alitretinoin is the approved systemic therapy for HE in Europe [1], which confirms the reliability of our repurposing analyses. Interestingly, alitretinoin targeted fewer genes in our (V)HE network compared to the other retinoids, isotretinoin and tretinoin. According to our results, tretinoin, together with cyclosporine and silicon dioxide, was one of the drugs that reversed the expression of the most genes in the VHE transcriptome: 11 out of the 52 VHE DEGs. Based on our findings, isotretinoin and tretinoin may hold potential for treating VHE. Topical tretinoin and adapalene and oral isotretinoin are licensed for acne vulgaris [41,54] but have not been investigated in eczema, to our knowledge. A retinoid that was not included as a drug repurposing candidate is the oral acitretin, which has been approved for treating psoriasis [41] and may be considered as an off-label treatment in hyperkeratotic HE [1]. Acitretin was linked to the retinoid acid receptor genes in DrugBank [17]. These genes were not included in our network, which explains why acitretin was not a drug repurposing candidate.

### 4.3. Immunomodulating Drugs

Glucocorticosteroids, immunosuppressants cyclosporine, methotrexate, azathioprine, sulfasalazine, and the more selective therapies tofacitinib and dupilumab have emerged as our most promising candidates for drug repurposing in (V)HE. Glucocorticosteroids have anti-inflammatory effects. Topical corticosteroids are already recommended as first-line treatment in HE for a short duration, and intermittent long-term use may be considered. Short-term oral corticosteroids are suggested in acute and severe inflammation [1]. Moreover, the broad systemic immunosuppressants, cyclosporine, methotrexate, and azathioprine, are currently suggested as off-label treatment options to treat severe HE refractory or contra-indicated to alitretinoin [1]. In a retrospective study, it was observed that 68% of VHE patients (*n* = 66) achieved a clinical improvement of over 50% with cyclosporine compared to 63% of all HE patients (*n* = 102) [55]. This positive effect in VHE could be underpinned with our results, as cyclosporine targeted the most genes of our (V)HE network. Furthermore, the immunosuppressant sulfasalazine, approved for inflammatory bowel diseases and rheumatoid arthritis, was included in our most promising drug repurposing candidate list and is sometimes used off-label in other dermatological diseases (e.g., psoriasis, alopecia areata, and pemphigus) due to its broad anti-inflammatory effects [56]. Importantly, the use of broad immunosuppressants often leads to early discontinuation due to adverse effects, which highlights the need for more targeted therapies [55,57,58,59].

Another immunosuppressant repurposing candidate is tofacitinib, a Janus Kinase (JAK)-1/3 inhibitor approved for rheumatoid arthritis, psoriasis arthritis, and ulcerative colitis. JAK-inhibitors, with their broad impact on multiple inflammatory pathways, offer potential in diverse immune-mediated diseases. Efficacy of tofacitinib was demonstrated in psoriasis in phase III and long-term extension trials [60,61]. JAK-1/3 inhibition leads to inhibition of Th1-, Th2-, and Th17-associated cytokines [62,63], which seem to play a role in the pathogenesis of HE [1]. However, systemic tofacitinib may elevate the risk of deep vein thrombosis and pulmonary embolisms, particularly in patients already at a high risk [64].

Contrary to our expectations, no JAK-inhibitors other than tofacitinib emerged as drug repurposing candidates. Pan-JAK inhibitors (topical delgocitinib [65] and oral gusacitinib [66]) and JAK1-inhibitors (oral baricitinib [67] and upadacitinib [68]) demonstrated promising efficacy in chronic HE in recent RCTs or case-reports. An explanation could be that our (V)HE network did not include JAK genes because we selected DEGs with stringent DEG-criteria [13]. JAK3 was highly upregulated in lesional VHE skin vs. healthy control skin with a fold change of 3.11 but did not reach the threshold of >500 averaged normalized counts [13]. Thus, besides the JAK1/3-inhibitor tofacitinib, another oral JAK3/tyrosine kinase-inhibitor, ritlecitinib, might be an interesting treatment option for (V)HE. Ritlecitinib has been approved by the U.S. Food and Drug Administration for the treatment of severe alopecia areata [69,70]. Furthermore, the topical application of the pan-JAK inhibitor delgocitinib cream was effective and well tolerated in chronic HE with a distinct advantage, i.e., that its localized and limited topical application minimizes the safety concerns inherent to systemic administration of JAK-inhibitors [71].

Dupilumab is a human monoclonal antibody binding to the interleukin (IL)-4 receptor, inhibiting IL-4 and IL-13. Dupilumab is registered for the skin diseases moderate-to-severe AD and prurigo nodularis [41,72]. Dupilumab has shown efficacy in atopic HE (prospective observational study, *n* = 72 [73]; a retrospective multicenter study, *n* = 84 [74]; and a phase 3 placebo-controlled RCT, *n* = 133 [75]) and in VHE (phase IIb placebo-controlled RCT, *n* = 29 [76]). In a retrospective observational study, hyperkeratotic HE seemed less likely to show improvement compared to all other subtypes [77]. The long-term use, its efficacy and tolerance make dupilumab a promising therapy option for chronic VHE, but larger RCTs with classification of subtypes and longer duration are needed.

### 4.4. Hormones

Interestingly, estradiol and progesterone emerged as (V)HE drug repurposing candidates. Bilateral ovariectomy in mice resulted in reduced skin barrier function, decreased skin recovery after acute disruption, increased irritant contact dermatitis [78], and downregulated epidermal proteins, filaggrin and involucrin [79]. Furthermore, post-menopausal stratum corneum contained lower ceramide levels, whereas estradiol treatment enhanced ceramides [80]. Overall, estrogen may improve skin barrier homeostasis [78,81], while progesterone seems to impair skin barrier homeostasis and delay recovery [82,83,84]. Thus, estradiol seems a more favorable repurposing candidate than progesterone for (V)HE.

### 4.5. Antihistamines

Systemic antihistamines targeting the histamine 1 receptor, like fexofenadine, are often prescribed for treatment of chronic HE. A cross-sectional study showed that antihistamine use in 1255 chronic HE patients was related to disease severity, pruritus, and comorbidity with atopic diseases. However, there is no supporting evidence that more severe HE should be treated with antihistamines and that antihistamines could reduce itch [85].

### 4.6. Statins

Statins, known for their cholesterol-lowering properties, exhibit pleiotropic anti-inflammatory and immunomodulatory effects [86]. Consequently, statins were investigated in atopic and dermatological diseases. Statin use in adults with asthma was associated with a reduced risk of asthma exacerbations and improved clinical outcomes in a retrospective study [87]. Furthermore, a systematic review demonstrated that lipid-lowering drugs, particularly oral and topical statins, can significantly improve psoriasis lesions [88]. The role of oral statins in vitro and mouse model studies in vitiligo showed beneficial effects, but clinical human trials did not identify a significant improvement in vitiligo symptoms. This discrepancy might be due to a low statin dose as a higher dose of systemic statins can cause cutaneous adverse reactions [41]. Exploring topical statin application offers a promising alternative, allowing for potentially higher local concentrations with minimal adverse effects [89]. A double-blind placebo-controlled RCT demonstrated that adding topical atorvastatin 5% cream to betamethasone 1% ointment was more effective in treating chronic HE (*n* = 44) after 10 days compared to betamethasone ointment plus a vehicle cream (*n* = 44), as evidenced by significantly greater reductions in HE severity and itching and improvements in quality of life [90]. Thus, statin use as an adjuvant therapy may be beneficial in the treatment of (V)HE patients, but more research is needed.

### 4.7. Antineoplastic Drugs

Various antineoplastic drugs (tamoxifen, erlotinib, etoposide, and fluorouracil) were included in our most promising drug repurposing list. Tamoxifen, a non-steroidal estrogen receptor modulator, before and after type 1 sensitization of mice was demonstrated to inhibit allergic responses with a reduction of allergen-specific Ig levels and reduced allergen-induced dermatitis [91]. A psoriasis patient responded favorably to oral administration of tamoxifen. As systemic tamoxifen can cause serious adverse effects, topical tamoxifen formulations were developed that showed preclinical efficacy on psoriasis-like lesions in mice [92].

Erlotinib, an epidermal growth factor receptor (EGFR)-inhibitor, shows potential by regulating keratinocyte proliferation and terminal differentiation through EGFR signaling. Overactive EGFR signaling plays a role in different keratinizing disorders with palmoplantar keratoderma (PPK), like pachyonychia congenita (most significant in the category ‘Disease’ in our functional enrichment results) [93,94,95]. Other EGFR-inhibitors were also included in our drug repurposing list but had no drug ranking score >0. Several case series reported that treatment with oral or topical erlotinib improved PPK symptoms [93,94,95,96]. A clinical subtype of HE, called hyperkeratotic HE, is characterized by the presence of palmar hyperkeratotic plaques that resembles PPK symptoms. EGFR-signaling was enriched among our genes in the (V)HE network. In contrast to keratinizing disorders, lesional AD skin exhibited decreased expressions levels of EGFR [97]. Consequently, EGF treatment, and thus EGFR-signaling stimulation, might be protective in AD as it reduced skin inflammation of AD-like lesions in mice, enhanced skin barrier function, and improved Th1, Th2, and Th17 immune responses [98,99,100]. Furthermore, topical EGFR-inhibitor application in mouse models with contact hypersensitivity, before introducing the contact allergen, led to increased skin inflammation compared to vehicle-treated groups [101]. These findings underline the complexity and limited knowledge of EGFR signaling in different skin diseases.

### 4.8. Vitamins and Minerals

Niacinamide, an amide form of vitamin B3 (niacin), is involved in the biosynthesis of stratum corneum lipids (e.g., ceramids) and can have antimicrobial and anti-inflammatory effects [102]. There is growing evidence according to case reports and small RCTs that oral or topical niacinamide could be beneficial in treating several skin diseases, like bullous pemphigoid, linear IgA bullous dermatosis, acne vulgaris, and AD [103,104]. Niacinamide as an add-on therapy in skin diseases could be advantageous, but well-powered RCTs are needed.

Micronutrient zinc is topically used in many dermatological diseases due to its anti-inflammatory effects on adaptive and innate immunity and its interference with keratinocyte differentiation and proliferation [105]. Zinc deficiency, seen in genetic disorders (e.g., acrodermatitis enteropathica), is also reported in inflammatory skin diseases, such as alopecia, autoimmune bullous dermatoses, AD, and VHE [105,106,107,108]. Systematic reviews suggest that oral zinc suppletion may contribute to clinical improvement in acne vulgaris, hidradenitis suppurativa, diaper dermatitis, and AD, although its evidence is not so strong [107,108]. An intra-individual right-to-left RCT demonstrated that 0.05% clobetasol + 2.5% zinc sulphate cream exhibited significant superior efficacy in chronic HE (*n* = 47) compared to clobetasol alone, without specifying subtypes [109].

### 4.9. Antibiotics and Antibacterials

Antibiotics (rifampicin, framycetin) and antibacterials (silvernitrate) were identified as drug repurposing options for (V)HE. Chronic HE and its disease severity compared to healthy controls are associated with skin microbiome dysbiosis, which is characterized by reduced bacterial α-diversity and increased relative abundance of *S. aureus*. These findings were observed in all subtypes of HE, including VHE [110]. Framycetin could target *S. aureus* [41]. However, antibiotics have the potential to lead to antibiotic resistance [111].

### 4.10. Strengths and Limitations

A strength is that our study incorporates both transcriptomics and genomics data, leading to a more comprehensive drug repurposing list. In addition, the most significant pathways according to the functional enrichment analyses among our network were expected [13], and provided confidence in the validity of our input gene list and our method of network construction. Overall, our study introduced an innovative and systematic approach to identify drug repurposing opportunities, to our knowledge. Furthermore, the most promising drug repurposing candidates were systematically prioritized using a transparent custom-build drug ranking system, thereby preventing the risk of confirmation bias [112].

A limitation is that stringent criteria were used for the selection of DEGs from our previous published VHE study [13], which ensured validity of the genes included in our network but may have led to potential false negatives. Furthermore, while our study aims to identify drug repurposing opportunities for VHE, it is essential to acknowledge that the GWAS of HE [16] was based on self-reported physician-diagnosed HE in the general population, encompassing all clinical subtypes instead of only VHE. This could potentially have led to misclassification resulting in some false positives and/or negatives in our (V)HE network. Thus, despite including genes from two molecular layers, not all genes from current genomics and transcriptomics studies may have been retrieved with high validity. Other molecular layers, such as epigenomics or proteomics, can further help in the identification of important mechanisms [15]. Moreover, it is important to note that our full drug repurposing candidate list is quite long and diverse, and interpretation of these findings must be approached with caution. Therefore, we have incorporated our drug ranking system and defined the most promising drugs. For example, an intravenous antithymocyte immunoglobulin was included in our drug repurposing candidate list, which might induce VHE as a cutaneous adverse effect [113], although thymocyte immunoglobulin was not identified as the most promising according to our drug ranking system. A suggestion for future research is to replicate our findings through cross-validation with other databases and to investigate whether our most promising drug candidates according to our drug ranking system could be truly applicable and effective in (V)HE.

## 5. Conclusions

Our (V)HE network, based on transcriptomic VHE and genomic HE data, provided potential drug repurposing opportunities for the treatment of (V)HE through drug–gene interactions. Among the 123 identified drugs, we prioritized the most promising based on our drug ranking system. While many show beneficial efficacy in skin diseases, variations in effectiveness, safety, and utility in the context of severe (V)HE must be acknowledged. Future studies should investigate the clinical efficacy of these drugs. Furthermore, exploring transcriptome and drug repurposing studies of different HE subtypes could mark an initial step towards achieving more personalized and thus optimally tailored treatments for HE patients.

## Figures and Tables

**Table 1:** Drug ranking criteria to select the most promising drug repurposing candidates for treatment of (vesicular) hand eczema.

Bonus Criteria	Points
Drugs with immunomodulating efficacy in inflammatory/auto-immune diseases (in a completed phase 2 interventional trial and at least an ongoing phase 3 trial) ^1,2^	1
Drugs with immunomodulating efficacy in inflammatory/auto-immune skin diseases (in a completed phase 2 interventional trial and at least an ongoing phase 3 trial) ^1,2^	2
Drugs that target > 3 genes of our (V)HE network	1
Topical application or possibility of compounding into a topical application ^3^	1
Part of World Health Organization (WHO) list of essential medicines 2023 ^4^	1
The price of the drug is low (50–500 EUR/year) ^5^	1
The price of the drug is very low (<50 EUR/year) ^5^	2
**Penalty Criteria**	**Points**
No Anatomical Therapeutic Chemical (ATC)-code ^6^	−2
Not available for order ^3^	−1
Unclear direction of effect on genes	−1
0.1–1% chance on grade 3–5 adverse events: severe, life-threatening, death ^5^	−1
Practical issues ^3,5,6^	−1
Severe practical issues ^3,5,6^	−2
The price of the drug is not available (in addition to the drug not being available for order) or is very expensive (>20,000 EUR/year) ^5^	−1

^1^ Data retrieved from ClinicalTrials.gov [36]. ^2^ Data retrieved from PubMed [37]. ^3^ Data retrieved from KNMP Kennisbank [39]. ^4^ Data retrieved from the WHO model list of essential medicines 2023 [38]. ^5^ Data retrieved from Farmacotherapeutisch Kompas [41]. ^6^ Data retrieved from WHO ATC index [40]. (V)HE: (vesicular) hand eczema.

**Table 2:** Ranking of drug repurposing candidates for treatment of (vesicular) hand eczema with a score > 0.

Score	Drug	Medicine Group (with ATC-Code)	Target Gene(s)
6	Calcitriol	Vitamin D and analogues (A11CC04/D05AX03)	*BIRC3*, *C1S*, *S100A8*, *SERPINB3*, *SNTB1*, *TNC*
6	Estradiol	Hormones (G03CA03)	*KRT17*, *KRT6A*, *KRT6B*, *KRT6C*, *MSMB*, *PARP9*
5	Tretinoin	Retinoids (D10AD01)	*BIRC3*, *FABP7*, *HAS3*, *KRT17*, *KRT6A*, *LAPTM5*, *LYZ*, *S100A8*, *S100A9*, *SNTB1*, *TNC*
5	Atorvastatin	Lipid modifying drugs (C10AA05)	*DPP4*
5	Methotrexate	Immunosuppressants (L04AX03)	*AADAC*, *CD1A*, *MMP12*, *S100A9*
5	Prednisolone	Glucocorticoids (H02AB06/D07XA)	*MMP12*
5	Adapalene	Retinoids (D10AD03)	*JUN*
4	Isotretinoin	Retinoids (D10BA01)	*CHI3L2*, *KRT16*, *KRT17*, *KRT6A*, *MSMB*
4	Sulfasalazine	Immunosuppressants (A07EC01)	*NFKB1*
4	Zinc	Vitamins and minerals (A12CB/D02AB)	*S100A8*; *S100A9*; *C1R*; *C1S*; *KRT16*; *KRT6A*; *SERPINA3*
4	Acetaminophen	Analgesics (N02BE01)	*CD1A*, *DPP4*, *IL37*, *LCE3A*, *LCP1*, *MSMB*, *PARP9*, *SERPINA3*, *TYMP*
4	Acetylsalicylic acid	Analgesics/antithrombotic drugs (N02BE01/B01AC06)	*SERPINA3*, *TYMP*
4	Azathioprine	Immunosuppressants (L04AX01)	*DPP4*, *MMP12*
4	Dexamethasone	Glucocorticoids (H02AB02/D07AB)	*IL4R*, *MSMB*
4	Fexofenadine	Antihistamines (R06AX26)	*CCL22*
3	Lidocaine	Analgesics (N01BB02/D04AB01)	*EGFR*
3	Diazepam	Benzodiazepine derivatives (N05BA01)	*TNC*
3	Cyclophosphamide	Antineoplastic agents— Nitrogen mustard analogues (L01AA01)	*BIRC3*
3	Cyclosporine	Immunosuppressants—calcineurin inhibitor (L04AD01)	*C1R*, *C1S*, *DPP4*, *LAPTM5*, *LYZ*, *MT4*, *PARP9*, *S100A8*, *TNC*, *S100A9*, *SERPINA3*
3	Cytarabine	Antineoplastic agents-pyrimidine analogues (L01BC01)	*CDH3*, *FABP7*
3	Diclofenac	NSAID (M01AB05/D11AX18)	*MMP12*
3	Methylprednisolone	Glucocorticoids (H02AB04/D07AA)	*MMP12*
3	Rifampicin	Antibiotics (J04AB02)	*IL37*
3	Selenium	Vitamins & minerals (A12CE)	*BIRC3*
3	Silver nitrate	Antibacterial (D08AL01)	*MT4*
3	Simvastatin	Lipid modifying agents (C10AA01)	*IL4R*
3	Tofacitinib	Immunosuppressants—selective (L04AA29)	*KRT16*
3	Valproic acid	Anti-epileptics (N03AG01)	*C1S*, *CHP2*, *DPP4*, *FABP7*, *SERPINB3*
2	Dupilumab	Interleukin-inhibitor (D11AH05)	*IL4R*
2	Framycetin	Antibiotics (D09AA)	*CXCR4*
2	Irbesartan	Angiotensin II receptor blockers (C09CA04)	*JUN*
2	Niacin	Vitamins and minerals (A11)	*NNMT*
2	Vildagliptin	Blood glucose lowering drugs-DPP4 inhibitors (A10BH02)	*DPP4*
2	Alitretinoin	Retinoids (D11AH0)	*KRT17*, *KRT6A*, *S100A8*
2	Bexarotene	Retinoids (L01XF03)	*KRT17*
2	Indomethacin	NSAID (M01AB01)	*BIRC3*
2	Nicotine	Drugs used in nicotine dependence (N07BA01)	*LTF*
2	Progesterone	Hormones (G03DA04)	*KRT17*, *SPRR2B*, *TNC*
2	Rosuvastatin	Lipid modifying agents (C10AA07)	*PI3*
2	Tamoxifen	Antineoplastic agents–anti-estrogens (L02BA01)	*SERPINA3*
2	Nadroparin	Antithrombotic drugs (B01AB06)	*FOS*
1	Erlotinib	Antineoplastic agents-Epidermal growth factor receptor inhibitors (L01EB02)	*EGFR*
1	Saxagliptin	Blood glucose lowering drugs—Dipeptidyl peptidase 4 (DPP4) inhibitors (A10BH03)	*DPP4*
1	Etoposide	Antineoplastic agents—podophyllotoxin derivatives (L01CB01)	*BIRC3*
1	Fluorouracil	Antineoplastic agents—pyrimidine analogues (L01BC02)	*SNTB1*, *TYMP*
1	Silicon dioxide	No ATC-code, used as a compound in medicine	*AADAC*, *C1R*, *C1S*, *KRT17*, *LCN2*, *LOR*, *PARP9*, *SERPINA3*, *SERPINB3*, *SERPINB4*, *TMEM173*

## Data Availability

The data presented in this study are available within the article and Supplementary Materials.

## Supplementary Materials

The following supporting information can be downloaded at https://www.mdpi.com/article/10.3390/pharmaceutics16040476/s1: Figure S1: Splicing-Quantitative Trait Locus (QTL) (A) and expression-QTL (B) genes associated with hand eczema identified in skin tissue based on the GTEx database; Figure S2: The distribution of node degrees for the constructed (vesicular) hand eczema network. *Y*-axis shows the number of nodes with a certain calculated degree of centrality; Figure S3: Reactome diagram of the keratinization pathway with nodes involving our (vesicular) hand eczema genes highlighted in purple; Figure S4: Immune pathways involving our (vesicular) hand eczema genes highlighted in purple; Figure S5: Reactome diagram of the antimicrobial peptides pathway with nodes involving our (vesicular) hand eczema genes highlighted in purple; Table S1: sQTL (A) and eQTL (B) lookup results of the (V)HE GWAS locus in skin based on GTEx database; Table S2: Node centrality parameters calculated for VHE combined network through network analysis with Cytoscape. Table S3: Centrality parameters calculated for interactions in the (V)HE combined network through network analysis with Cytoscape. Table S4: Functional enrichment results of the 52 VHE DEGs (A) and the 3 HE QTLs (B) in Reactome pathways; Table S5: Functional enrichment of the combined (V)HE network, including 52 VHE genes, 3 HE genes, and 23 linker genes, in Reactome pathways; Table S6: Functional enrichment results of the 11 identified modules within the combined (V)HE network; Table S7: Functional enrichment results of the combined (V)HE network in functional categories from 12 different databases using STRING; Table S8: Functional enrichment results of the combined (V)HE network in functional categories from 12 different databases using STRING; Table S9: Drugs targeting (V)HE genes based on the DrugBank database; Table S10: Results of the pharmaco-transcriptomic lookups based on the DrugBank database; Table S11: Ranking system of drugs targeting (V)HE genes based on the DrugBank database; Table S12: Ranking system of results of the pharmaco-transcriptomic lookups based on the DrugBank database; Table S13: Total ranking score of drug repurposing candidates for treatment of (V)HE with and without pricing criteria.
