# Supplementary material for: Transcriptomics- and Genomics-Guided Drug Repurposing for the Treatment of Vesicular Hand Eczema
Source: Pharmaceutics. 2024 Mar 30;16(4):476. doi: 10.3390/pharmaceutics16040476 (PMC11054470; doi:10.3390/pharmaceutics16040476)
Supplement: Supplementary file 1 [file pharmaceutics-16-00476-s001.zip › Supplementary Figures.pdf]

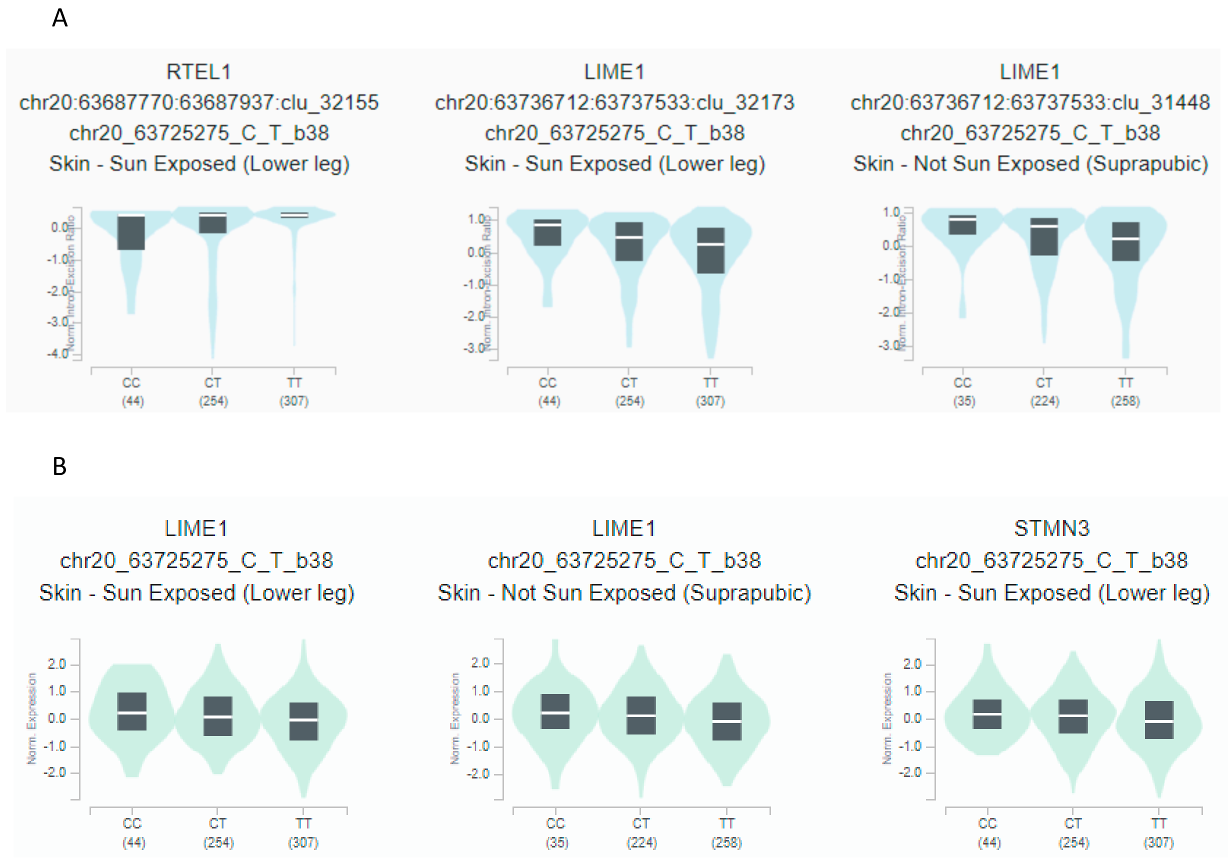

Supplementary Figure S1. Splicing-Quantitative Trait Locus (QTL) A) and expression-QTL (B) genes associated with hand eczema identified in skin tissue based on the GTEx database.

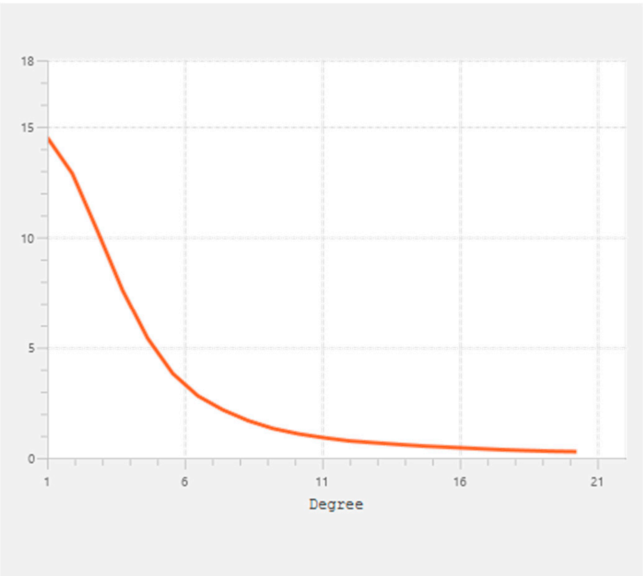

Supplementary Figure S2. The distribution of node degrees for the constructed (vesicular) hand eczema network. Y-axis shows the number of nodes with a certain calculated degree of centrality.

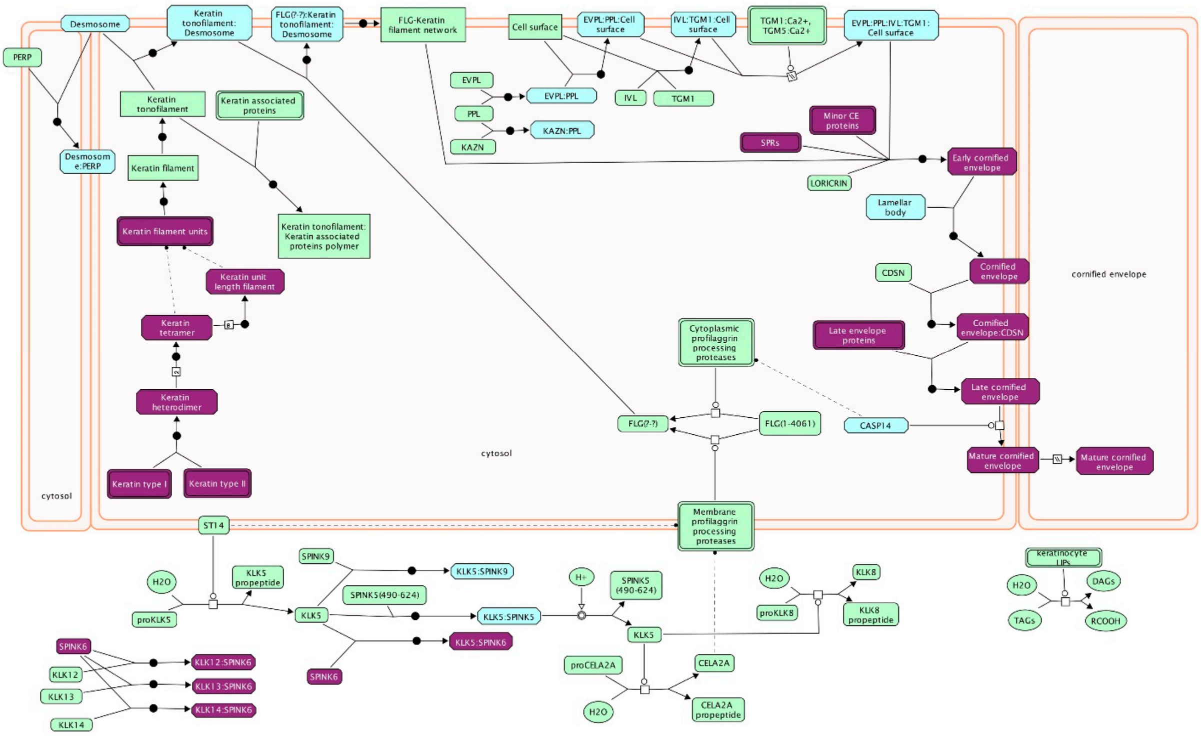

Supplementary Figure S3. Reactome diagram of the keratinization pathway with nodes involving our (vesicular) hand eczema genes highlighted in purple.

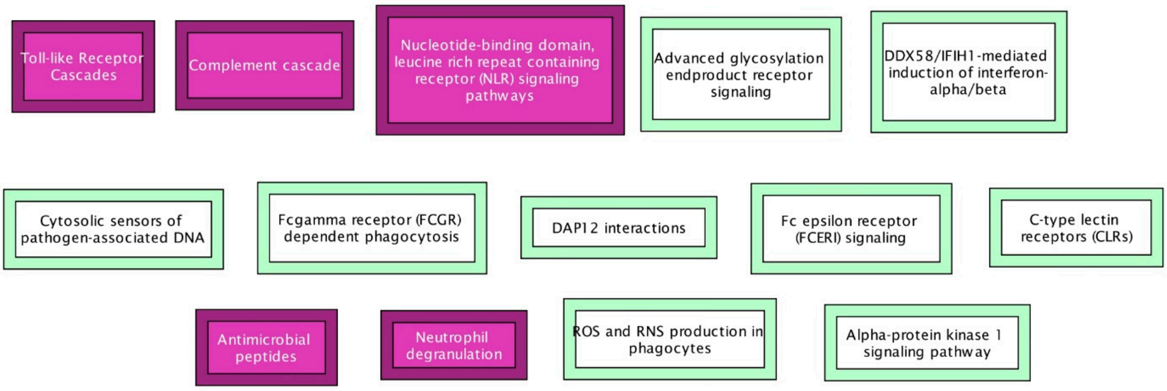

Supplementary Figure S4. Immune pathways involving our (vesicular) hand eczema genes highlighted in purple.

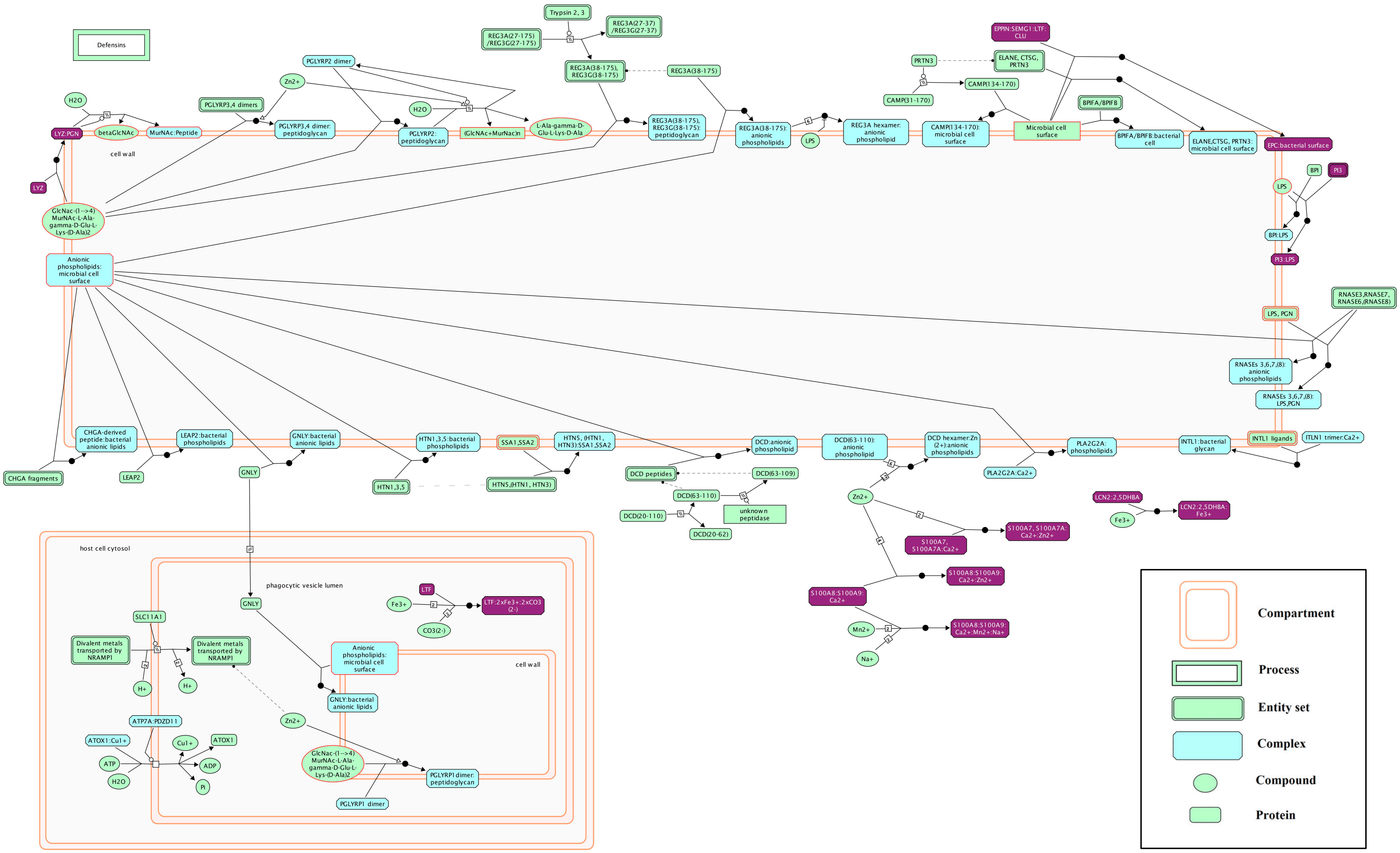

Supplementary Figure S5. Reactome diagram of the antimicrobial peptides pathway with nodes involving our (vesicular) hand eczema genes highlighted in purple.
